# MXene‐Stabilized VS_2_ Nanostructures for High‐Performance Aqueous Zinc Ion Storage

**DOI:** 10.1002/advs.202401252

**Published:** 2024-04-12

**Authors:** Liping Zhang, Yeying Li, Xianjie Liu, Ruping Yang, Junxiao Qiu, Jingkun Xu, Baoyang Lu, Johanna Rosen, Leiqiang Qin, Jianxia Jiang

**Affiliations:** ^1^ Flexible Electronics Innovation Institute (FEII) Jiangxi Key Laboratory of Flexible Electronics Jiangxi Science and Technology Normal University Nanchang 330013 China; ^2^ Laboratory of Organic Electronics (LOE) Department of Science and Technology Linköping University Norrköping 60174 Sweden; ^3^ Department of Physics, Chemistry and Biology (IFM) Linköping University Linköping 58183 Sweden

**Keywords:** aqueous zinc‐ion batteries, heterogeneous layered structure, structural stability, Ti_3_C_2_T_z_ MXene, VS_2_

## Abstract

Aqueous zinc‐ion batteries (AZIBs) based on vanadium oxides or sulfides are promising candidates for large‐scale rechargeable energy storage due to their ease of fabrication, low cost, and high safety. However, the commercial application of vanadium‐based electrode materials has been hindered by challenging problems such as poor cyclability and low‐rate performance. To this regard, sophisticated nanostructure engineering technology is used to adeptly incorporate VS_2_ nanosheets into the MXene interlayers to create a stable 2D heterogeneous layered structure. The MXene nanosheets exhibit stable interactions with VS_2_ nanosheets, while intercalation between nanosheets effectively increases the interlayer spacing, further enhancing their stability in AZIBs. Benefiting from the heterogeneous layered structure with high conductivity, excellent electron/ion transport, and abundant reactive sites, the free‐standing VS_2_/Ti_3_C_2_T_z_ composite film can be used as both the cathode and the anode of AZIBs. Specifically, the VS_2_/Ti_3_C_2_T_z_ cathode presents a high specific capacity of 285 mAh g^−1^ at 0.2 A g^−1^. Furthermore, the flexible Zn‐metal free in‐plane VS_2_/Ti_3_C_2_T_z_//MnO_2_/CNT AZIBs deliver high operation voltage (2.0 V) and impressive long‐term cycling stability (with a capacity retention of 97% after 5000 cycles) which outperforms almost all reported Vanadium‐based electrodes for AZIBs. The effective modulation of the material structure through nanocomposite engineering effectively enhances the stability of VS_2_, which shows great potential in Zn^2+^ storage. This work will hasten and stimulate further development of such composite material in the direction of energy storage.

## Graphical abstract

The stable 2D heterogeneous layered composite films are designed by mixing VS_2_ and MXene in a simple one‐step process. Composite films can be used as both cathode and anode of aqueous zinc‐ion batteries (AZIBs). Furthermore, it exhibits impressive stability with 97% capacity retention after 5000 cycles and high operation voltage (2.0 V) for the flexible Zn‐metal free in‐plane AZIBs.

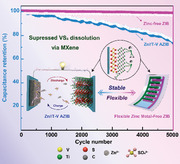

## 1 Introduction

With the increasing awareness of environmental concerns, the development of green energy sources such as solar, wind, and biomass has increased over recent years. However, the sustainable and clean alternatives to fossil fuels have a major drawback, which is the inability to maintain a constant and continuous supply of electricity due to their intermittent nature. Therefore, the development of a safe, green, economical, and efficient electrochemical energy conversion system that can accumulate the electrical energy obtained from renewable resources and then integrate it into the power grid has become a critical necessity of our society.^[^ ^1^ ^]^ In recent years, rechargeable aqueous zinc‐ion batteries (AZIBs) have emerged as excellent candidates for grid‐scale energy storage systems due to their intrinsic advantages, including high safety, environmental benignity, specific power, and reversibility. Additionally, they boast non‐toxicity and low costs.^[^ ^2^ ^]^ Therefore, in order to promote the commercialization of AZIBs, various materials have been designed to obtain robust and high‐efficiency host materials. These encompass vanadium‐based compounds,^[^ ^3^ ^]^ manganese‐based oxides,^[^ ^4^ ^]^ organic molecules and polymers,^[^ ^5^ ^]^ Prussian blue analogues,^[^ ^6^ ^]^ and transition metal dichalcogenides.^[^ ^7^ ^]^

Among them, vanadium‐based compounds have gained extensive attention owing to the rich redox chemistry of V^5+^/V^3+^ couple that enables high specific capacities.^[^ ^8^ ^]^ Specifically, vanadium disulfide (VS_2_) has a vanadium layer between two sulfur layers to form a sandwich structure, and it has an interlayer spacing of 5.76 Å. The large interlayer spacing and the weak electrostatic interaction between VS_2_ and divalent Zn^2+^ due to the low electronegativity of S^2−^ can achieve faster ion‐diffusion kinetics^[^ ^9^ ^]^ and a theoretical study^[^ ^10^ ^]^ has revealed that VS_2_ mono layers could be able to absorb three layers of ions, which contributes to a large theoretical capacity as high as 1397 mAh g^−1^. These factors collectively make VS_2_ a promising host material for high‐performance AZIBs. However, the vanadium‐sulfur bond binding force is relatively weak, and the crystal structure is easily destroyed during the charge and discharge process, resulting in poor cycle stability. In addition, low electronic conductivity further limits its performance in AZIBs. Therefore, in order to further improve the electrochemical activity and cycle durability of VS_2_‐based electrodes, structural engineering strategies are used to composite VS_2_ nanosheets with conductive materials^[^ ^11^ ^]^ or introduce pillar ions.^[^ ^7a^ ^]^ These strategies not only endow the prepared hybrids with excellent electronic/ionic conductivity, large specific surface area, and high electrochemical stability, but also significantly enhance the storage capacity of zinc ions.

MXene,^[^ ^12^ ^]^ an emerging large family of 2D layered transition metal carbides and nitrides where the by far most explored compound is Ti_3_C_2_T_z_ (T refers to surface terminations), has been widely explored for, e.g., photodetectors,^[^ ^13^ ^]^ sensors,^[^ ^14^ ^]^ and energy storage,^[^ ^15^ ^]^ and conversion.^[^ ^16^ ^]^ Due to their nanolaminate microstructure, excellent electrical conductivity, good hydrophilicity, and sufficient surface chemistry, MXenes are considered to be very promising materials for the development of high‐performance electrodes for multivalent ion batteries. In addition, the MXene‐based electrodes with stable layered structures may improve long‐term cycling behavior, which is crucial for high‐performance AZIBs. Still, due to low capacity and operating voltage, few pure MXenes have been used as cathode materials. However, if the conductive 2D layered structure of MXene can be exploited together with highly active materials, there may be an increase in the layer spacing and a potentially high and fast Zn^2+^ storage capacity achieved.

Although the ZIBs are considered to be strong contender for next generation of safe and environment‐friendly energy storage systems, the commercialization of ZIBs has been plagued by issues such as zinc dendrite growth and gas evolution on zinc metal anodes during repeated charge/discharge processes, since these issues often result in low Coulombic efficiency and short lifespan for ZIBs. Lithium‐ion batteries, which are also limited by the application of metal anodes, have recently proposed the concept of “anode‐free” metal batteries for the first time using inactive substrates as the current collectors. Based on this, “anode‐free” Zn batteries^[^ ^17^ ^]^ were also accomplished recently in water‐based electrolytes. Since “anode‐free ZIB” is a new concept that was proposed in recent two years, there is little works have been performed in this field.^[^ ^18^ ^]^ At the same time, the currently reported anode‐free ZIBs generally show unsatisfactory electrochemical properties, especially cycling stability due to unsatisfactory anode metal plating–stripping efficiency and/or narrow potential window of existing electrolytes.^[^ ^19^ ^]^

Herein, we enhance the electrochemical performance of VS_2_ in AZIBs through a composite engineering strategy. The free‐standing and flexible VS_2_/Ti_3_C_2_T_z_ composite electrodes were fabricated by facile mixing colloidal solution of VS_2_ and Ti_3_C_2_T_z_. This optimized heterogeneous layered structure is favorable to promote the aqueous electrolyte penetration, enhance the electronic/ionic conductivity, improve the structural stability, and provide abundant zinc ion storage active sites. Thus, the resulting VS_2_/Ti_3_C_2_T_z_ composite electrodes showed excellent zinc‐ion storage capacity as either a cathode or anode in AZIBs. In Zn//VS_2_/Ti_3_C_2_T_z_ batteries, the VS_2_/Ti_3_C_2_T_z_ cathode exhibits a high specific capacity (285 mAh g^−1^ at 0.2 A g^−1^) and very good cycling performance (76.7% capacity retention after 5000 cycles at 2 A g^−1^), as well as high energy density of 415 Wh kg^−1^.More importantly, the flexible Zn‐metal free in‐plane VS_2_/Ti_3_C_2_T_z_//MnO_2_/CNT battery delivers high operation voltage, outstanding long‐term cycling stability (with a capacity retention of 97% after 5000 cycles at a current density of 2 A g^−1^), and desirable mechanical durability, showing great potential for wearable device fields. In addition, this work will provide new thinking in constructing high‐performance anode‐free ZIBs and promotes the development of ZIBs.

## 2 Results and Discussion

**Figure 1:**
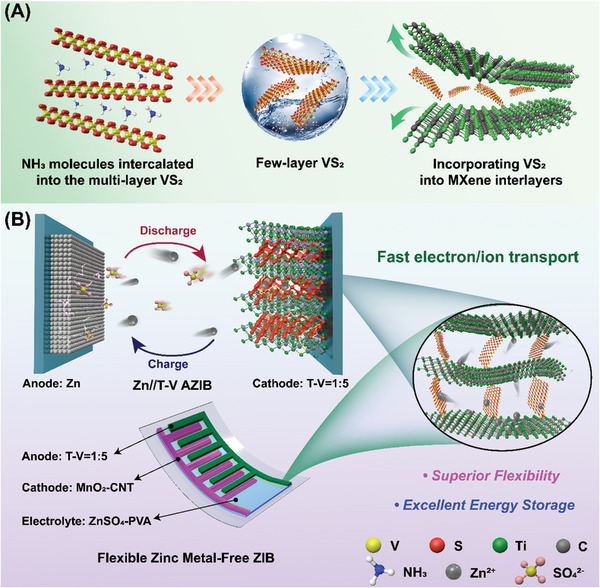
Preparation process of electrode materials and application of zinc storage devices. A) Fabricating steps of VS_2_/Ti_3_C_2_T_z_ composite films. B) Schematic structure of VS_2_/Ti_3_C_2_T_z_ composite films serving as a cathode/anode electrode for the construction of Zn//T‐V AZIBs and flexible zinc metal‐free AZIBs and illustration of the zinc storage mechanism of the devices.

**Figure 2:**
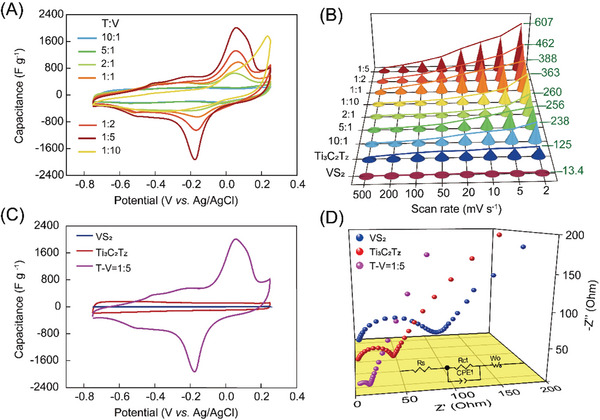
Electrochemical performance of the prepared films in 1 M ZnSO_4_ in a three‐electrode configuration. A) CV curves of VS_2_/Ti_3_C_2_T_z_ composite films with different mass ratios at a scan rate of 2 mV s^−1^. B) Rate performance of pristine Ti_3_C_2_T_z_, VS_2_ films, and VS_2_/ Ti_3_C_2_T_z_ composite films at different scan rates. C) Comparison of CV curves for Ti_3_C_2_T_z_, VS_2_, and T‐V = 1:5 films at a scan rate of 2 mV s^−1^. D) Electrochemical impedance spectroscopy of Ti_3_C_2_T_z_, VS_2_, and VS_2_/Ti_3_C_2_T_z_ composite films. (inset: Equivalent circuit mode corresponding to the Nyquist plots).

The Ti_3_C_2_T_z_ MXene is prepared by the classic MILD method and the VS_2_ colloidal nanosheets are growing under hydrothermal conditions (see details in the Experimental Section, Supporting Information). The tailored heterogeneous layered free‐standing composite electrodes were prepared by simple one‐step physical mixing of colloidal solutions of Ti_3_C_2_T_z_ and VS_2_ through vacuum filtration, as schematically illustrated in **Figure** **1**A. Taking advantage of the 3D open framework of the VS_2_/Ti_3_C_2_T_z_ composite films, the reversible adsorption of Zn^2+^ and SO_4_^2‐^ during the electrochemical reaction process can be achieved (Figure 1B). During the discharge process, Zn^2+^ is adsorbed and embedded in the VS_2_/Ti_3_C_2_T_z_ cathode. Meanwhile, SO_4_^2−^ moves toward the Zn anode which maintains the balance of charges in the battery. During the charging process, Zn^2+^ is separated from the VS_2_/Ti_3_C_2_T_z_ cathode and transferred to the Zn anode to form zinc, and anions return from the Zn anode to ZnSO_4_. The composite films with different ratios were prepared to explore their optimal properties. Pristine Ti_3_C_2_T_z_ MXene and VS_2_ were also prepared for comparison. For pristine VS_2_, it is a very brittle film that is difficult to peel off from the filter membrane. However, all composite films are flexible free‐standing films (Figure S1, Supporting Information), indicating that the introduction of MXene can greatly improve the mechanical properties of the composite films. The electrochemical properties of the as‐prepared composite films are evaluated in 1 M ZnSO_4_ aqueous solution at different scan rates (Figures S2–S4, Supporting Information). **Figure** **2**A displays the cyclic voltammetry (CV) curves of the VS_2_/Ti_3_C_2_T_z_ composite films with different mass ratios at the scan rate of 2 mV s^−1^. It is indicated that the composite electrodes with a mass ratio of Ti_3_C_2_T_z_:VS_2_ = 1:5 (referred to as T‐V = 1:5) exhibit a maximum capacitance of 607 F g^−1^ (Figure 2B). As shown in Figure 2C, the T‐V = 1:5 displays a significant improvement in electrochemical performance compared to the electrodes before the composite. The presence of a pair of distinct redox peaks at 0.06 V and ‐0.18 V in the CV curves of T‐V = 1:5 is attributed to the stepwise Zn^2+^ intercalation and deintercalation processes. This shows that MXene greatly improved the electron and ion transport properties of the composite film. A similar conclusion can be obtained from Nyquist plots (Figure 2D; Figure S5, Supporting Information). The intrinsic charge transfer kinetics are established by recording the electrochemical impedance spectroscopy curves within a frequency range of 100 kHz–0.1 Hz and a possible equivalent circuit diagram is also shown as the inset of Nyquist plots. The equivalent circuit consists of equivalent series resistance (Rs) and charge transfer resistance (Rct), together with a slope attributed to Warburg impedance (Zw), corresponding to Zn‐ion diffusion.^[^ ^20^ ^]^ Considering the same testing system used in the present work, the Rs is mostly related to the electrode conductivity.^[^ ^21^ ^]^ It is shown that the value of Rs for T‐V = 1:5 film (1.053 Ω) is lower than the pristine VS_2_ (1.927 Ω) and Ti_3_C_2_T_z_ (1.68 Ω) films, indicating a higher conductivity of the VS_2_/MXene composite films and the reduced value of Rs would boost the power density.^[^ ^22^ ^]^ For the charge transfer kinetics, Rct is a key factor. The T‐V = 1:5 composite film displayed a much smaller Rct (10.28 Ω) than VS_2_ (111.5 Ω) and Ti_3_C_2_T_z_ (44.09 Ω) electrodes. The small Rct would be beneficial to high rate capability,^[^ ^23^ ^]^ which explains the excellent rate performance of the VS_2_/MXene composite films. In the high‐frequency region, the T‐V = 1:5 composite film shows a rather small charge transfer resistance with an invisible semicircle, indicating fast electron transfer performance. Furthermore, in the mid‐frequency region, the composite film exhibits a smaller Warburg impedance than Ti_3_C_2_T_z_, showing a rather small ion transport resistance of the heterogeneous structure, indicating that the electrolyte ions have fast ion diffusion and wetting properties in the composite electrodes.

**Figure 3:**
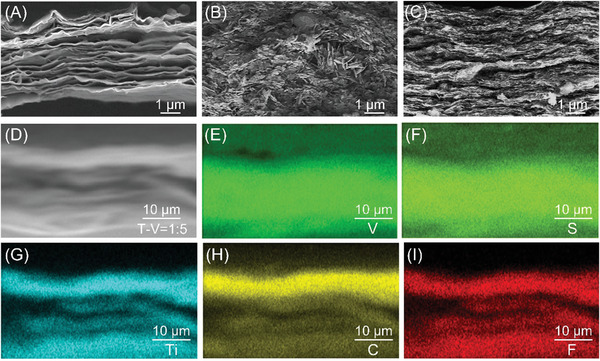
Physical characterizations. Cross‐sectional SEM image of A) Ti_3_C_2_T_z_, B) VS_2_, C) T‐V = 1:5. SEM image (D) of T‐V = 1:5 with the EDS mapping of E) V, F) S, G) Ti, H) C, I) F.

Structural characterization was performed to explore the impact of composite electrode structure on performance. The cross‐section scanning electron microscopy (SEM) images of the Ti_3_C_2_T_z_, VS_2_, and composite films are shown in **Figures** **3**A**–C** and S6 (Supporting Information). The pristine Ti_3_C_2_T_z_ films exhibit well‐aligned stacked MXene sheets (Figure 3A). However, VS_2_ exhibits disorderly stacked nanosheets (Figure 3B), which would lead to poor mechanical properties. The T‐V = 1:5 composite films displayed a clear heterogeneous layered structure in which Ti_3_C_2_T_z_ and VS_2_ are alternately arranged (Figure 3C). The VS_2_ nanosheets tend to adhere to the surface of Ti_3_C_2_T_z_, changing the disordered accumulation state of pristine VS_2_. The SEM‐EDS mapping images (Figure 3D–I) indicate the homogeneous distribution of Ti_3_C_2_T_z_ and VS_2_ in the composite films.

**Figure 4:**
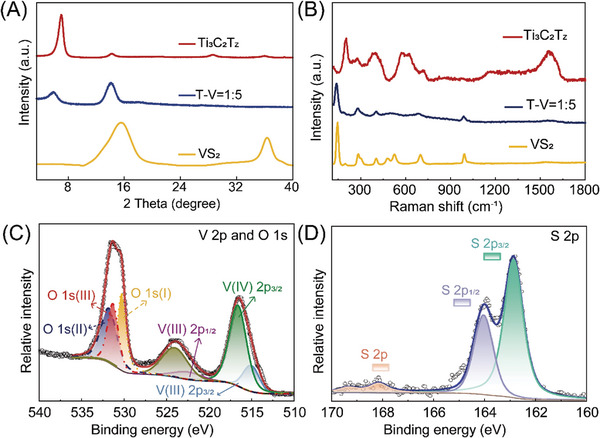
Physical characterizations. A) XRD patterns and B) Raman spectra of Ti_3_C_2_T_z_, VS_2_ and T‐V = 1:5 films. XPS spectra of the VS_2_: V 2p and O 1S (C), S 2p (D).

The X‐ray diffraction (XRD) patterns (**Figure** **4**A) of VS_2_ show a major peak at 15.49°, which originates from the (001) diffraction peak of VS_2_ nanosheets. In addition, the obvious (002) peak of Ti_3_C_2_T_z_ appears at ≈7.0°. After composite, the (002) peak of Ti_3_C_2_T_z_ and (001) peak of VS_2_ both move to a lower angle (Ti_3_C_2_T_z_ move to 5.8°, VS_2_ move to 14.1°), demonstrating the expanded interspace between layers after compounding (Figure S7, Supporting Information). At the same time, the decrease in the relative peak intensity of Ti_3_C_2_T_z_ indicates that the heterogeneous layered structure of the composite film effectively inhibits the close packing of MXene. The Raman spectrum of VS_2_ (Figure 4B) shows six characteristic peaks located at 141.2, 192.1, 281.5, 406.3, 688.5, and 993.4 cm^−1^, which are assigned to the rocking and stretching vibrations of V‐S bonds or their combination.^[^ ^24^ ^]^ For Ti_3_C_2_T_z_, the Raman peaks located at 202, 278, and 398 cm^−1^ represent in‐plane (*E*_g_) vibrations of surface groups attached to titanium atoms, and the peaks located at 576, 620, and 720 cm^−1^ are assigned mostly to carbon vibrations (both E_g_ and A_1g_). The Raman spectrum of the T‐V = 1:5 composite films was also studied. It mainly showed the peaks of VS_2_, which may be due to the fact that the main part of the composite material is VS_2_. In addition, the decrease in relative Raman peak intensity of the composite film compared with pristine VS_2_ indicates that the interaction between Ti_3_C_2_T_z_ and VS_2_ limits the vibration of the V‐S bond after composite formation. The X‐ray photoelectron spectroscopy (XPS) of VS_2_ was measured, and the corresponding curves are shown in Figure 4C,D.^[^ ^25^ ^]^ The high‐resolution V 2p spectra of VS_2_ display the doublet peaks located at 524.0 and 516.5 eV, corresponding to the 2p_1/2_ and 2p_3/2_ peaks of V(IV) and V (III), respectively.^[^ ^26^ ^]^ The S 2p spectrum presents two signals at 164.0 and 162.9 eV that can be allocated to the S 2p_1/2_ and S 2p_3/2_. The weak peak of S 2p at higher binding energy (168.2 eV) may originate from oxidized S, which can also be explained by the presence of O 1s signals. The oxygen groups on the VS_2_ will help to increase its hydrophilicity thus facilitating the penetration of aqueous electrolyte and the transport of hydrated Zn^2+^. The corresponding elements of VS_2_ and Ti_3_C_2_T_Z_ both have XPS signals in the XPS spectra of the T‐V = 1:5 free‐standing film (Figure S8, Supporting Information). In addition, the ratio of V and Ti is close to the 1:5 ratio of Ti_3_C_2_T_Z_ and VS_2_ in the composite film (Table S1, Supporting Information). The above results show that the heterogeneous structure enhances the accessibility between electrode materials and electrolyte, and Ti_3_C_2_T_z_ acts as a firm conductive scaffold, to alleviate aggregation and structure collapse of VS_2_ during repeated charge/discharge processes. As a result, the T‐V = 1:5 electrode is expected to show excellent electrochemical properties for ZIBs.

**Figure 5:**
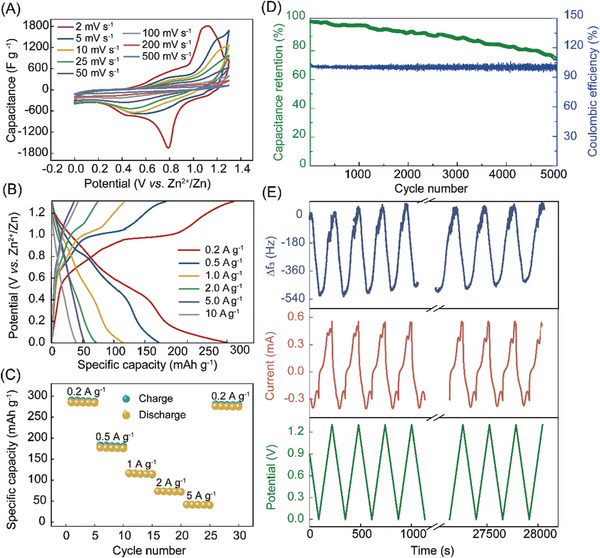
Electrochemical performance of Zn//T‐V = 1:5 batteries cycling in a range of 0–1.3 V. A) CV curves at different scan rates. B) Charge and discharge curves at the current density from 0.2 to 10 A g^−1^. C) Rate performance. D) Cycling life performance. E) Time dependence of the EQCM‐D parameters: applied potential, current, and frequency.

The electrochemical properties of the T‐V = 1:5 composite cathodes are evaluated by employing zinc foil as an anode and an aqueous electrolyte (1 M ZnSO_4_) adsorbed with a glass fiber separator. **Figure** **5**A shows the CV curves of T‐V = 1:5 composite electrodes at different scan rates, in which the two pairs of obvious redox peaks located at 0.78/1.12 (tested at 2 mV s^−1^) are attributed to the gradual intercalation and deintercalation process of Zn^2+^, especially at low scan rate. The galvanostatic charge/discharge profile of the T‐V = 1:5 composite is shown in Figure 5B, which shows two pairs of potential plateaus agree well with the CV results. The rate performances for T‐V = 1:5 composite electrodes from 0.2 to 5 A g^−1^ are presented in Figure 5C. At the low current of 0.2 A g^−1^, the cell exhibited an initial discharge capacity of 285 mAh g^−1^. Due to limitations in reaction kinetics, the capacity gradually decreases with increasing current density. However, the reversible capacity can quickly recover to 276.5 mAh g^−1^ once the current density is back to 0.2 A g^−1^, demonstrating excellent high‐rate tolerance. Cycle stability of the T‐V = 1:5 composite was evaluated at a current density of 2 A g^−1^. As shown in Figure 5D, the T‐V = 1:5 composite with a high‐capacity retention of 74.3% and coulombic efficiency of ≈100% after 5000 cycles, is superior to the most recently reported aqueous Zn storage materials based on transition‐metal dichalcogenides (TMDs). The excellent performance is largely attributed to the enhancement of the stability of the layered structure by Ti_3_C_2_T_z_, which avoids phase transformation, collapse, or degradation of the VS_2_ layered structure during repeated insertion/extraction of hydrated cations. Herein, the T‐V = 1:5 electrode after 6000 cycles were characterized with XRD and SEM. Figure S9a (Supporting Information) show the XRD comparison of the T‐V = 1:5 electrode before and after long cycling tests. By comparing the characteristic peaks, it is observed that the peaks shift to lower angles (from 5.94° to 4.52°, from 14.61° to 13.69°) (Figure S9a, Supporting Information). This shift is primarily attributed to the increased interlayer spacing resulting from the trapped zinc ions during cycling. Meanwhile, the electrode maintains the characteristic peaks before cycling and retains its layered structure after cycling (Figure S9b,c, Supporting Information)). To further explore the reason for the high stability of the composite films, a method to analyze charge carriers in the electrochemical process, namely in situ electrochemical quartz crystal microbalance (EQCM) techniques, was performed. This technology reflects the mass change of the sensor by measuring the vibration frequency (the change in mass is inversely proportional to the change in frequency). EQCM results consisting of applied potential, current, and frequency are shown in Figure 5E. The frequency tracks well with the corresponding CV curves, in which step changes in responses are correlated to the redox peaks of the composite film. The potential starts from 0.88 V (open circuit voltage) to 0 V and the EQCM results indicate that the mass increases during the discharging process, which is related to the insertion of Zn^2+^. Subsequently, the electrode mass decreases during charging at a potential higher than 0.4 V, which represents the start of Zn^2+^ desorption. When the potential reaches 1.3 V, the frequency almost returns to the initial position, which indicates that zinc ions perform almost fully reversible insertion/extraction in the T‐V = 1:5 composite film. To further monitor the mass changes of the electrode during cycling, long‐term EQCM was performed. After 100 cycles, the electrode still showed good mass reversibility, which shows that the layered heterostructure of the composite film effectively improves the ion transmission efficiency of the composite film and avoids the decrease in stability caused by the “dead zinc” phenomenon that occurs in pristine VS_2_.

**Figure 6:**
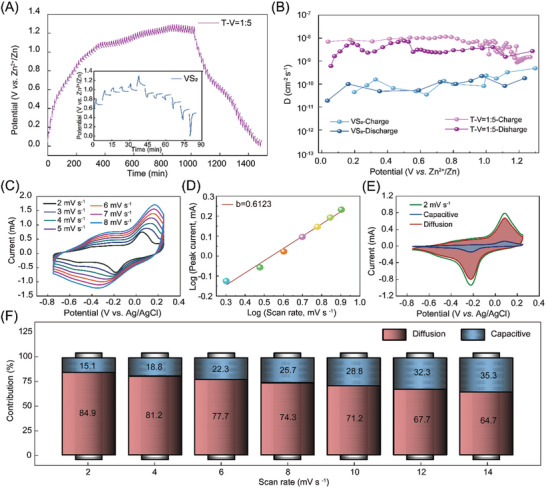
Quantitative capacitive analysis of Zn‐ion storage behavior. A) Galvanostatic intermittent titration technique (GITT) profiles. B) Zn‐ion diffusion coefficient calculated based on GITT. C) CV curves of T‐V = 1:5 at various scan rates from 2 to 8 mV s^−1^. D) The corresponding log (peak current) versus log (scan rate) plots at the redox peak for the T‐V = 1:5 cathode. E) CV profile at 2 mV s^−1^ showing the capacitive contribution (shaded area) to the total current. F) The contribution ratios of the capacitive and diffusion‐controlled charge at different scan rates in the T‐V = 1:5 cathode.

Galvanostatic intermittent titration technique (GITT) measurements were further conducted to calculate the Zn‐ion diffusion rate of the VS_2_/MXene composite films in AZIB. The GITT analysis was performed after the first cycle to eliminate the interference from the side reactions in the first cycle and reflect the intrinsic characteristics of the electrode materials. The value for D_Zn_^2+^ is computed from the GITT potential profile from Fick's second law:

(1) $$D\;=\frac{4L^{2}}{\Pi \tau }\;{\left(\frac{\Delta \mathrm{Es}}{\Delta Et}\right)}^{2}$$

where τ is the duration of the current pulse, L stands for the electrode thickness, τ, ΔEs, and ΔEt is obtained from the GITT curves. As can be seen from **Figures** **6**A,B, and S10 (Supporting Information), the GITT curves of the T‐V = 1:5 cathode shows obvious discharge or charge plateau during the electrochemical reaction, which agrees well with the GCD and CV results (Figure 6A). The calculated D_Zn2+_ values of the T‐V = 1:5 cathode is as high as 10^−9^ to 10^−8^ cm^2^ s^−1^, which is higher than the pristine VS_2_ (Figure 6B). This result shows the higher Zn‐ion diffusion kinetics and lower polarization with intensive interfacial interactions in the VS_2_/MXene cathode.^[^ ^27^ ^]^ Compared with the pristine VS_2_, the larger D _Zn2+_ values indicate faster electrochemical reaction kinetics, which is consistent with the EIS analysis. The high diffusion coefficient can directly reflect the rapid transport of Zn^2+^ in VS_2_/MXene composite electrode, further explaining the reason for obtaining good electrochemical performance. The high Zn ion diffusion coefficient is relevant to the heterogeneous electrode structure, in which Ti_3_C_2_T_z_ can provide the conductive skeleton for fast electron transport, while the nanostructure of VS_2_ provides a large specific surface and active site.

To gain insight into the charge storage mechanism of the T‐V = 1:5 composite, the CV curves are collected at scan rates from 2 to 8 mV s^−1^ (Figure 6C; Figure S11, Supporting Information). The capacity contribution from capacitance and diffusion behavior can be determined by the following power law: i = a*v* ^b^,^[^ ^28^ ^]^ where i is current, *v* is scanning rate, and a and b are variable parameters. Generally, the value of b ranges from 0.5 to 1. The b value of 1 indicates that the non‐faradaic reaction dominated through electrostatic phenomena and predominates in the electrode material storage (capacitance control), while the b value being 0.5 represents the charge being mainly stored through Faraday reactions (diffusion control). Through the linear relationship between log i and log *v* plots of the redox peak, as presented in Figure 6D, the b‐value was determined to be 0.612. It implies that the Zn^2+^ storage process of the T‐V = 1:5 is controlled by a synergistic interaction between the ionic diffusion and capacitance characteristics, yet the diffusion‐controlled behavior is the dominant process. Furthermore, the equation of i (V) = k_1_v + k_2_v^1/2^ is used to further quantify the contributions of capacitance and diffusion control to capacity.^[^ ^29^, ^30^ ^]^ As depicted in Figure 6E, the diffusion contribution dominantly accounts for 84.9% of the total capacity at a scan rate of 2 mV s^−1^. In addition, the contribution rate of the capacitive effect at different scan rates was quantitatively measured (Figure 6F). The diffusion contribution process occupies the dominant position in the total capacity and the contribution ratios of capacitive gradually increase with the increase of the scan rate.

**Figure 7:**
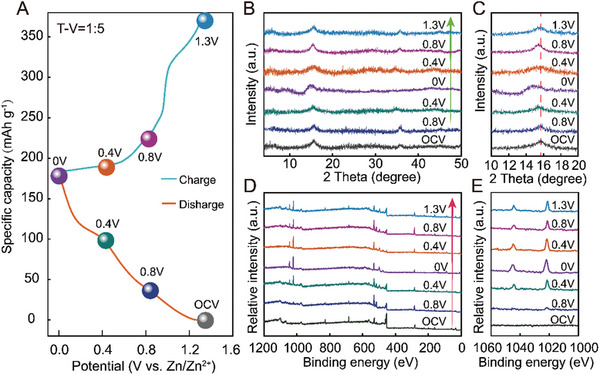
The Zn^2+^ storage mechanism of the T‐V = 1:5 electrodes in AZIBs. A) GCD curve of the T‐V = 1:5 electrode at 0.5 A g^−1^. The corresponding ex‐situ XRD patterns B,C) and ex‐situ XPS spectra (D)‐(E).

In order to gain a more comprehensive understanding of the Zn^2+^ storage mechanism of the T‐V = 1:5 electrodes, we have also performed the ex‐situ XRD and the ex‐situ XPS measurements during the charge/discharge cycle to track the structural evolution of the T‐V = 1:5 electrodes (**Figure** **7**). During the discharging process from OCV (0.9 V) to 0 V (Figure 7A), the (001) diffraction peak of VS_2_ shifts toward lower angles which is attributed to the Zn^2+^ intercalation (Figure 7B,C). Conversely, the (001) diffraction peak of VS_2_ moves back to the original position in the following charging process (from 0 to 1.3 V). This shows that the heterogeneous layered structure of the electrode maintains a high degree of structural reversibility during the migration of zinc ions. The ex‐XPS analysis was also used to investigate the behaviors of Zn elements during the discharge/charge process. As shown in Figure 7D,E, no Zn 2p signal was detected in the OCV state of the T‐V = 1:5 electrode, while the two peaks of Zn 2p (1021.9 and 1045.0 eV) were gradually enhanced during the discharge to 0 V, indicating that zinc ions were gradually inserted into the electrode maintains during the discharge process. After charging to 1.3 V, the intensity of the two peaks of Zn 2p is much lower than that of the fully discharged state, confirming that most zinc ions are extracted from the electrode. Small amounts of trapped Zn in the electrode might serve as “pillars” in the interlayers, further enhancing the layered structure of T‐V = 1:5 electrode during electrochemical cycles. Therefore, these results demonstrate the Zn ion intercalation is reversible, and the electrode structure remains highly reversible during the zinc ion intercalation process.

**Figure 8:**
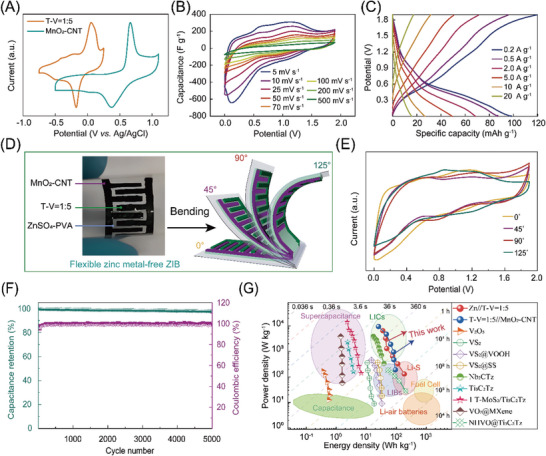
Electrochemical performance of flexible zinc metal‐free T‐V = 1:5//MnO_2_‐CNT ZIB. CV curves of A) MnO_2_‐CNT and T‐V = 1:5 films and B) the devices at different scan rates. C) Charge and discharge curves at the current density from 0.2 to 20.0 A g^−1^. D) Optical image and schematic diagram of the flexible zinc metal‐free ZIB. E) CV curves of the flexible zinc metal‐free ZIB device bent with different angles. F) Cycling life performance. G) Ragone plots of this work and reported counterparts.

Rechargeable zinc batteries have received increasing attention in recent years due to their safety, greenness, ease of manufacturing, and cost‐effectiveness. However, the practical application of zinc metal batteries is mainly hindered by the dendrite growth of zinc metal anodes, resulting in poor Coulombic efficiency, danger, and various side reactions. In addition, the use of zinc metal also hinders its use in flexible wearable devices. Here, a zinc metal‐free interdigitated planar flexible ZIB is constructed based on the T‐V = 1:5 composite as the anode, MnO_2_‐CNT as the cathode, and ZnSO_4_‐PVA as the gel electrolyte. The detailed characterization of MnO_2_‐CNT is shown in Figure S12 (Supporting Information). The charge balance between the two electrodes is achieved by controlling the thickness of the anode and cathode films (**Figure** **8**A). Finally, an operating potential window of up to 2.0 V is achieved for the flexible zinc metal‐free ZIBs, which is almost the widest voltage window reported to date for MXene‐based ZIBs (the widest voltage window is 2.4 V as Table S2, Supporting Information). The electrochemical performance of flexible zinc metal‐free ZIBs was investigated. The CV curves of full‐battery between 0 to 2.0 V at different scan rates are demonstrated in Figure 8B. At the scan rate of 2 mV s^−1^, two pairs of redox peaks located at 0.11/0.63 and 1.03/1.14 are attributed to T‐V = 1:5 composite and MnO_2_‐CNT electrodes. As the scan rate increases, the redox peaks are well maintained, indicating the excellent ion transport performance of the electrodes. Galvanostatic charge/discharge tests were performed within 0–2.0 V, which shows that two pairs of voltage plateaus agree well with the CV results. The initial capacity of the flexible zinc metal‐free ZIBs is 98.2 mAh g^−1^ at the current density of 0.2 A g^−1^ (Figure 8C). Interestingly, the quasi‐solid‐state zinc metal‐free interdigitated planar ZIB shows excellent flexibility, that is, there is minimal energy fading under different bending conditions (Figure 8D,E). More impressively, the zinc metal‐free ZIBs showed excellent long‐term cycle life with a capacity retention of 96.7% after 5000 cycles at 2 A g^−1^ (Figure 8F), which overwhelmingly outperforms the Zn//T‐V = 1:5 battery reported above. This shows that the zinc‐free metal structure is beneficial to improving the stability of the battery. In addition, the energy and power densities of different types of ZIBs, as well as a comparison with previously reported VS_2_ or MXene‐based ZIBs devices, are plotted in the Ragone plot as shown in Figure 8G. For Zn//T‐V = 1:5, a maximum energy density of 184.9 Wh kg^−1^ can be obtained at a specific power density of 130 W kg^−1^ and the energy density remains 35.6 Wh kg^−1^ at a high‐power density of 6500 W kg^−1^. For T‐V = 1:5//MnO_2_‐CNT, the energy density changes from 93.2 to 25.4 Wh kg^−1^ with a power density in the range of 190–9500 W kg^−1^. These values are better than ZIBs based on other materials, including transition metal oxides,^31^ sulfides,^32^ and carbides^33^ manufactured through various technologies.

## 3 Conclusion

In summary, we demonstrate an effective strategy for achieving high‐performance zinc ion storage by using tailored heterogeneous layer structured composite films as active electrodes, obtained from vacuum filtrated VS_2_ and Ti_3_C_2_T_z_ MXene based solution processable aqueous suspensions. The MXene intercalated VS_2_ nanolayer has typical nano size, expanded interlayer space, improved electronic and ionic conductivity, and MXene‐enhanced layered structure, showing good rate performance and excellent cycle stability, which is better than previous reports. The results show that the combination of MXene‐enhanced layered structure and nano‐sized VS_2_ can effectively eliminate the phase transition and subsequent structural collapse induced by hydrated Zn^2+^ insertion, which is important for obtaining long cycle life and high‐capacity utilization. More interestingly, composite films can serve as both a cathode and an anode in zinc‐ion batteries with different structures. As the cathode of Zn//T‐V = 1:5, the composite films achieved a high capacity of 285 mAh g^−1^ at 0.2 A g^−1^. Furthermore, as the anode of zinc metal‐free interdigitated planar flexible ZIB, the capacitance retention can reach 96.7% after 5000 cycles. All the results show that VS_2_/Ti_3_C_2_T_z_ composite film is a promising high‐performance zinc ion storage material. In addition, this work not only offers a new strategy for the design and fabrication of solution‐processable 2D heterogeneous layered structured composites, but also provides a method to develop aqueous batteries with low cost, high stability and safety, and potential for wearable energy supply devices.

## Conflict of Interest

The authors declare no conflict of interest.

## Supporting information

Supporting Information

## Data Availability

The data that support the findings of this study are available from the corresponding author upon reasonable request.
