# Supplementary material for: MXene‐Stabilized VS2 Nanostructures for High‐Performance Aqueous Zinc Ion Storage
Source: Adv Sci (Weinh). 2024 Apr 12;11(25):2401252. doi: 10.1002/advs.202401252 (PMC11220636; doi:10.1002/advs.202401252)
Supplement: Supplementary file 1 — Supporting Information [file ADVS-11-2401252-s001.pdf]

ADVANCED SCIENCE
Open Access

Supporting Information

for *Adv. Sci.*, DOI 10.1002/advs.202401252

MXene-Stabilized VS_2 Nanostructures for High-Performance Aqueous Zinc Ion Storage

Liping Zhang, Yeying Li, Xianjie Liu, Ruping Yang, Junxiao Qiu, Jingkun Xu, Baoyang Lu, Johanna Rosen, Leiqiang Qin* and Jianxia Jiang**

Supporting Information

MXene-stabilized VS_2 nanostructures for high-performance aqueous zinc ion storage

Liping Zhang, Yeying Li, Xianjie Liu, Ruping Yang, Junxiao Qiu, Jingkun Xu, Baoyang Lu*, Johanna Rosen, Leiqiang Qin*, and Jianxia Jiang*

L. Zhang, Y. Li, R. Yang, J. Qiu, J. Xu, B. Lu, J. Jiang

Flexible Electronics Innovation Institute (FEII), Jiangxi Key Laboratory of Flexible Electronics, Jiangxi Science and Technology Normal University, Nanchang 330013, China

L. Qin, J. Jiang, J. Rosen

Department of Physics, Chemistry and Biology (IFM), Linköping University, Linköping 58183, Sweden

X. Liu

Laboratory of Organic Electronics (LOE), Department of Science and Technology, Linköping University, Norrköping 60174, Sweden

1. **Experimental Section**

1.1. Materials Synthesis

1.1.1 Preparation of few-layer VS_2 suspension

Firstly, 0.3 mmol $Na_3VO_4 \cdot 12H_2O$ as a vanadium source and 15 mmol thioacetamide (TAA) as a sulfur source were dispersed into 40 ml distilled water and vigorously stirred at room temperature for 1 h. Then, the as-prepared homogeneous solution was transferred into a 50 mL Teflon-lined stainless-steel autoclave and maintained at 160 °C for 24 h and finally cooled down to room temperature. After that, the black precipitate, i.e., NH_3-intercalated $VS_2 \cdot NH_3$, was obtained by centrifugation and washed several times with distilled water. The obtained precursors were then dispersed in a round-bottomed flask with 30 mL water, protected by nitrogen gas, and ultrasonically stripped for 3h to exfoliate NH_3-intercalated $VS_2 \cdot NH_3$ into VS_2 ultrathin nanosheets. Finally, the suspension obtained above was centrifuged (5 min at 1500 rpm) to remove the precipitate, and the few-layer VS_2 suspension was obtained.

1.1.2. Preparation of few-layer $Ti_3C_2T_x$ suspension

Few-layer $Ti_3C_2T_x$ suspension was synthesized by etching Ti_3AlC_2 MAX powder in LiF/HCl hybrid etchant according to the previously reported route.[1] Typically, 1 g Ti_3AlC_2 powder was mixed with 1 g LiF and 15 mL 9 M HCl and stirred at 35°C for 48 hours. The acidic dispersion was washed by deionized H_2O until the pH of the supernatant turned to 7 via centrifugation (3 min at 6000 rpm). Finally, the few-layer $Ti_3C_2T_x$ suspension were obtained by centrifugation (30 min at 3500 rpm) to remove the precipitate.

1.1.3. Preparation of MnO_2-CNT suspension

1 g Mn_3O_4 powders·were added to 10 ml tetramethylammonium hydroxide solution (TMAOH, 25 wt. % in H_2O) and stirred at 80°C for 3 days. Afterwards the above reaction was washed with ethanol 5 times by centrifugation (6000 rpm for 2 min), then the resulting precipitate was washed three times with water, and then 30 ml of deionized water was added, shaken well, and sonicated for 20 minutes and the MnO_2 suspension were obtained by centrifugation at 6,000 rpm for 20 minutes. Finally, the suspension obtained above was ultrasonically mixed with CNT at a mass ratio of 1:1 to obtain the MnO_2-CNT suspension.

1.1.4. Fabrication of $Ti_3C_2T_x$/ VS_2 composite films

Few-layer $Ti_3C_2T_x$ and VS_2 suspensions were separately mixed in certain weight ratios, yielding a series of $Ti_3C_2T_x/VS_2$ suspensions with different mass ratios. Subsequently, each hybrid $Ti_3C_2T_x/VS_2$ suspension was filtered using a polypropylene membrane (Celgard 3501). After drying, the flexible $Ti_3C_2T_x/VS_2$ composite films were peeled easily from the membrane and labeled as T-V=10:1, T-V=5:1, T-V=2:1, T-V=1:1, T-V=1:2, T-V=1:5, and T-V=1: 10, respectively.

1.1.5. Fabrication of MnO_2-CNT composite films

Similarly, an appropriate amount of the prepared MnO_2-CNT suspension was taken for vacuum filtration, and the MnO_2-CNT membrane electrode was obtained by stripping from polypropylene membrane after drying.

1.2. Assembly of devices

1.2.1. Assembly of Zn // $Ti_3C_2T_x/VS_2$ batteries

For Zn // $Ti_3C_2T_x/VS_2$ batteries assembly, all used electrodes (Singed into a 6mm film with a hole punch) were the same as the previous tests. Systematically, Zn foil was used as the anode and the flexible $Ti_3C_2T_x/VS_2$ composite films were employed as the cathode in a two-

electrode system. The electrochemical performance of zinc battery was tested in 1M $ZnSO_4$ solution at room temperature.

1.2.2. Fabrication of metal-free interdigitated planar flexible ZIB

First, The $Ti_3C_2T_x/VS_2$ composite films and MnO_2-CNT films are pre-cut into interdigitated electrode shapes by laser, and then the two electrodes are assembled on a PET flexible substrate. 10 g poly (vinyl alcohol) (PVA, Mw: ~89000-98000) was completely dissolved in 20 mL 1 M $ZnSO_4$ at 80 °C. Then, the solution was kept at 80 °C without stirring. Second, the gel electrolyte was carefully casted on the interdigital pattern area and left in air for 1 h at room temperature to evaporate excess water.

1.3. In situ characterization

Electrochemical quartz crystal microbalance experiments. EQCM measurements were performed on a commercial Q-Sense Explore system (Biolin Scientific AB, Sweden). The composite electrode slurry was spray to titanium and gold coated AT-cut quartz crystal sensor chips, then dried at room temperature. The active material area of the sensor was 0.785 cm^2 (a circle with a diameter of 10 mm). EQCM was conducted during cyclic voltammetry (CV) tests on an electrochemical workstation (Autolab PGSTAT 302N).

1.4. Materials Characterizations

The crystal structures of samples were identified on a Bruker D2 Phaser X-ray diffractometer with Cu-Kα radiation (λ = 1.54178 Å). The morphology and microstructure of the different electrodes were investigated by means of field emission scanning electron microscopy (Zeiss Sigma 300) equipped with energy dispersive spectroscopy (EDS). XPS measurements were performed using monochromatic Al-Ka (1486.6 eV) radiation in a Kratos AXIS Ultra DLD system and a Scienta SES200 system. Raman spectra were collected on a Renishaw inVia at

532 nm. The EQCM-D were performed using E4 module, Q-Sense Explore (Biolin Scientific AB, Sweden).

1.5. Electrochemical measurements

All the electrochemical tests (cyclic voltammetry (CV), galvanostatic charge/discharge (GCD), electrochemical impedance spectroscopy (EIS) and galvanostatic cycling) were evaluated using an electrochemical workstation (VSP, Bio-Logic, France). The electrochemical performance of $Ti_3C_2T_x$, VS_2 and the $Ti_3C_2T_x/VS_2$ composite films were measured first with three-electrode Swagelok cells, where the $Ti_3C_2T_x/VS_2$ composite films (Singed into a 6mm film with a hole punch, respectively) were directly used as the working electrode, activated carbon was the counter electrode, an Ag/AgCl electrode was used as the reference electrode, and 1 M $ZnSO_4$ aqueous solution was the electrolyte. Electrochemical impedance spectroscopy was performed at open circuit potential, with a 10mV amplitude, and frequencies that ranged from 10 m Hz to 100 kHz.

The volumetric capacitance from the cyclic voltammetry data is determined from the following equation (1):

$$C = \frac{1}{\Delta V}\int \frac{jdV}{s} \quad (1)$$

C is the normalized capacitance (in units of F g^{-1}), *j* is the current density (in A g^{-1}), s is the rate (in V s^{-1}), V is the voltage (in V), ΔV is the voltage window (in V).

The specific capacitance from galvanostatic charge/discharge data is calculated from the following equation (2):

$$C = \frac{2It}{v} \quad (2)$$

where I and t represent the discharge current (mA) and time (h), respectively; *v* is the weight of one electrode or device.

The energy density of each device was obtained from the formula given in Equation (3):

$$E = \frac{1000}{2 \times 3600} C_v \Delta E^2 \qquad (3)$$

where E is the energy density in Wh g^{-1}, C_v is the capacitance obtained from galvanostatic charge/discharge curves using Equation (2) in F g-1 and ΔE is the operating voltage window in volts.

The power density of each device was calculated using the equation (4):

$$P = \frac{E}{t} \qquad (4)$$

where P is the power density in W cm^{-3} and t is the discharge time in hours.

2. Result Section

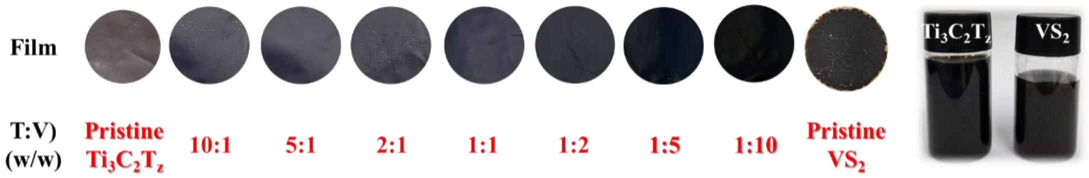

Figure S1. The image of the composite film for different $Ti_3C_2T_z$:VS_2 mass ratio and the colloidal solution of $Ti_3C_2T_z$ and VS_2.

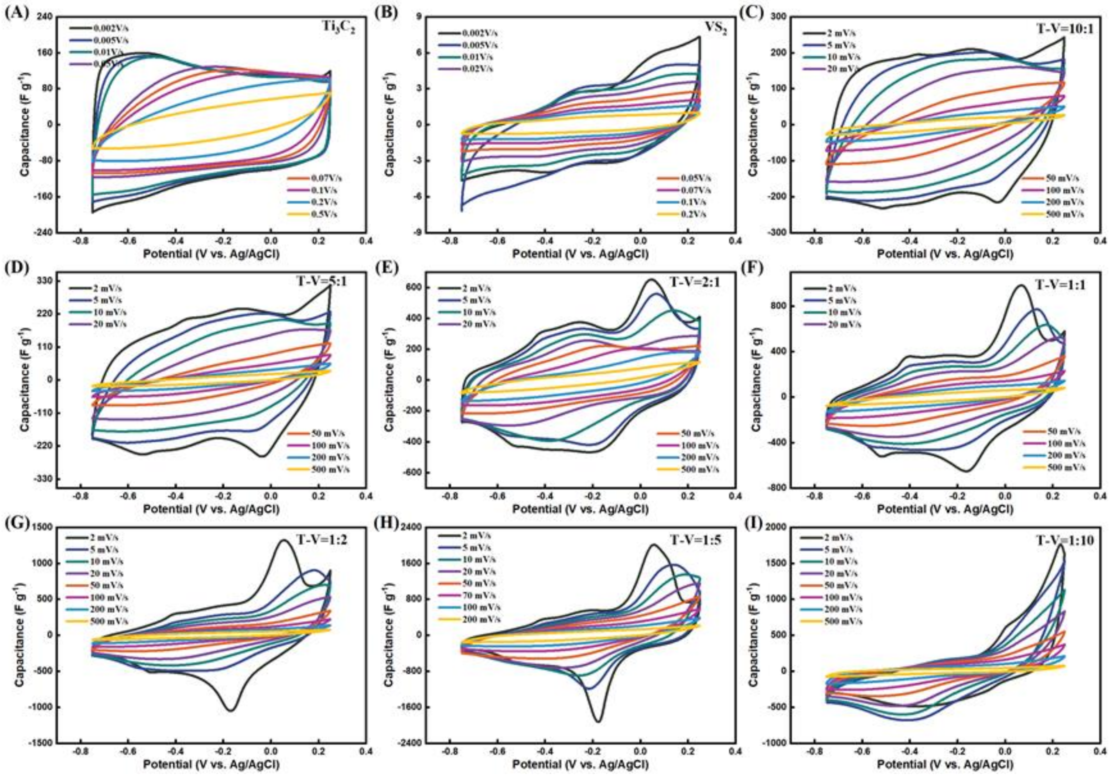

Figure S2. Cyclic voltammetry (CV) profiles of (A) $Ti_3C_2T_z$, (B) VS_2, (C) T-V=10:1, (D) T-V=5:1, (E) T-V=2:1, (F) T-V=1:1, (G) T-V=1:2, (H) T-V=1:5 and (I) T-V=1:10 obtained at different scanning rates.

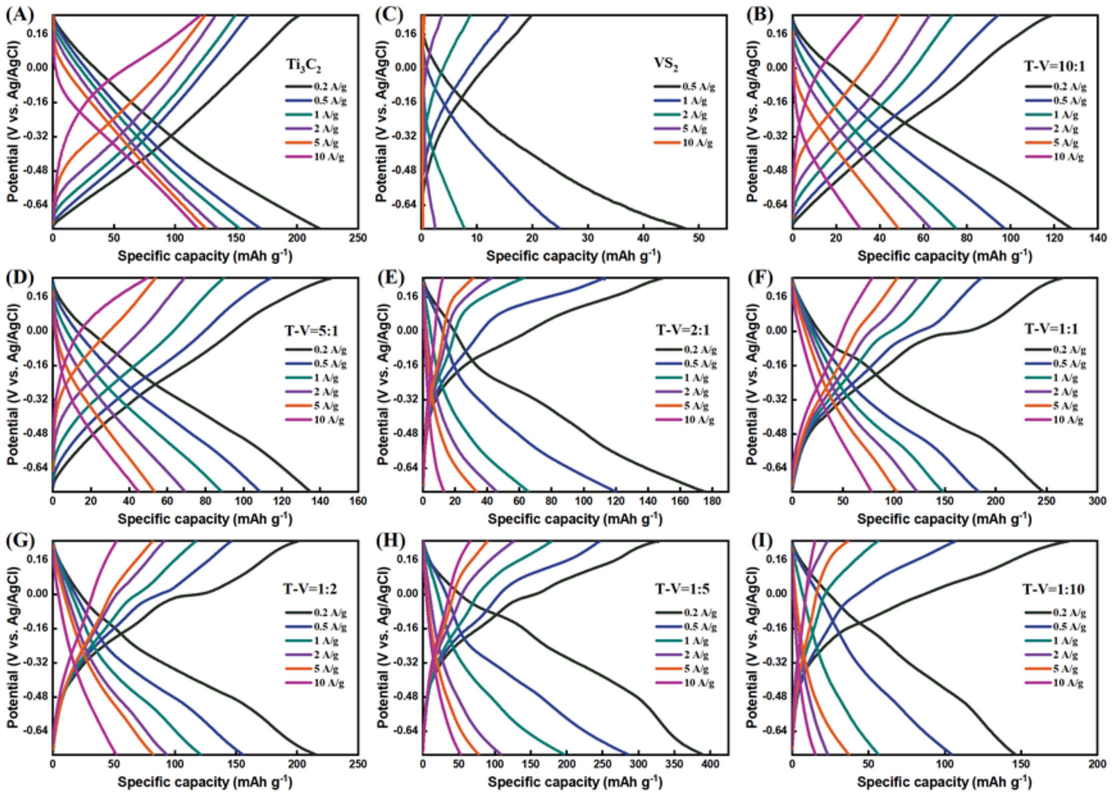

Figure S3. Charge and discharge curves of (A) $Ti_3C_2T_z$, (B) VS_2, (C) T-V=10:1, (D) T-V=5:1, (E) T-V=2:1, (F) T-V=1:1, (G) T-V=1:2, (H) T-V=1:5 and (I) T-V=1:10 obtained at different current density.

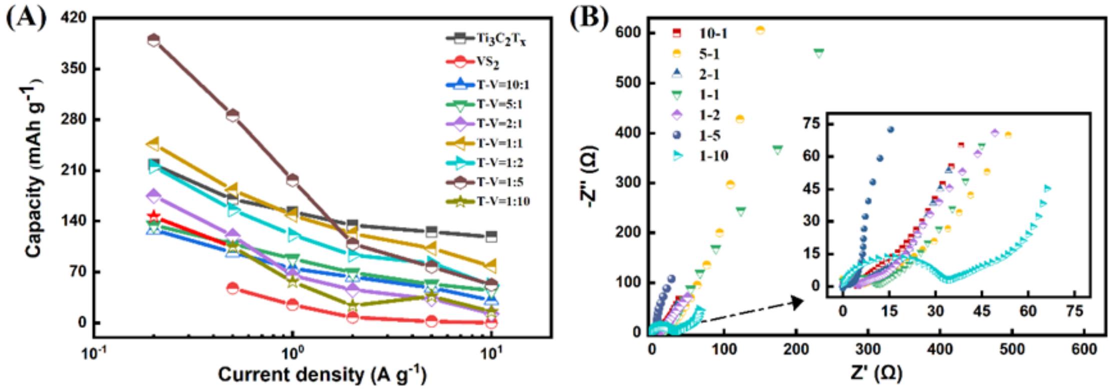

Figure S4. (A) Specific capacity of pristine $Ti_3C_2T_z$, VS_2 films and $Ti_3C_2T_z/VS_2$ composite films with different mass ratios at different current densities. (B) EIS characterization of different films.

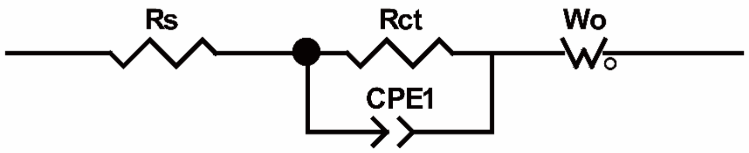

Resistance (Ω)	Rs	Rct
VS_2	1.927	111.5
$Ti_3C_2T_z$	1.68	44.09
T-V=1:5	1.053	10.28

Figure S5. The equivalent circuit for fitting EIS data and the obtained associated resistance value.

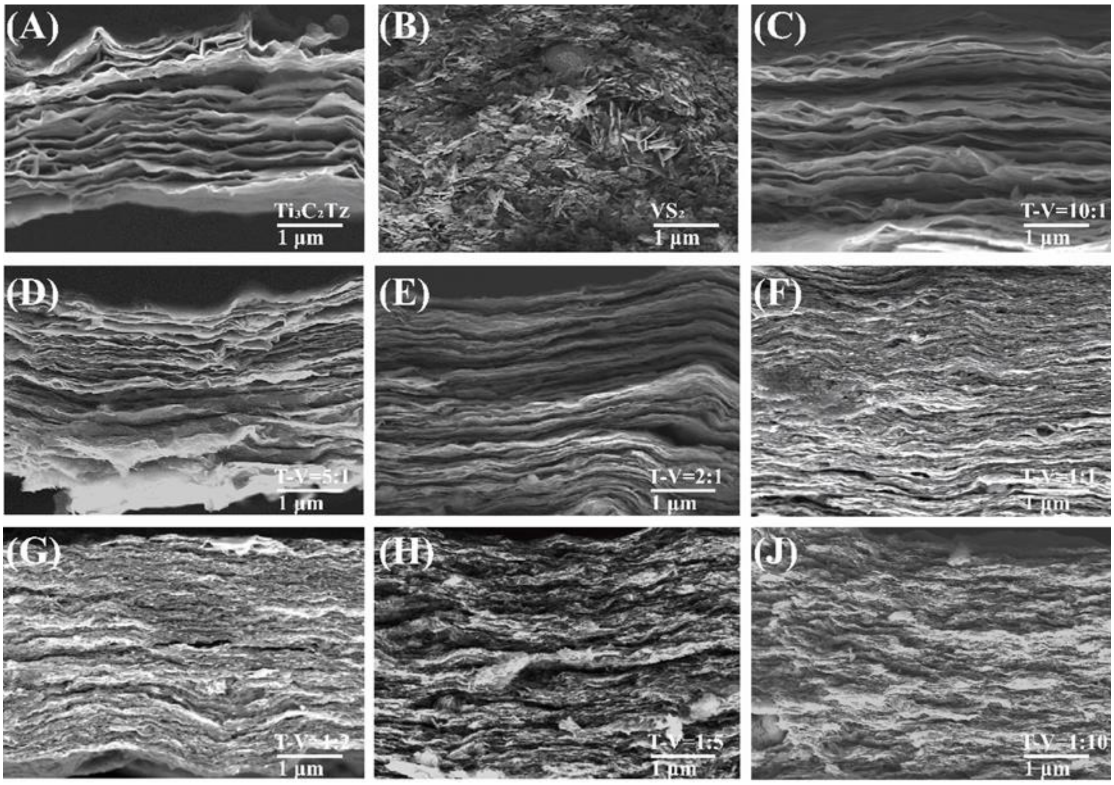

Figure S6. Cross-sectional SEM image of (A) $Ti_3C_2T_z$, (B) VS_2, (C) T-V=10:1, (D) T-V=5:1, (E) T-V=2:1, (F) T-V=1:1, (G) T-V =1:2, (H) T-V =1:5 and (I) T-V =1:10. Scale bars: 1 μm.

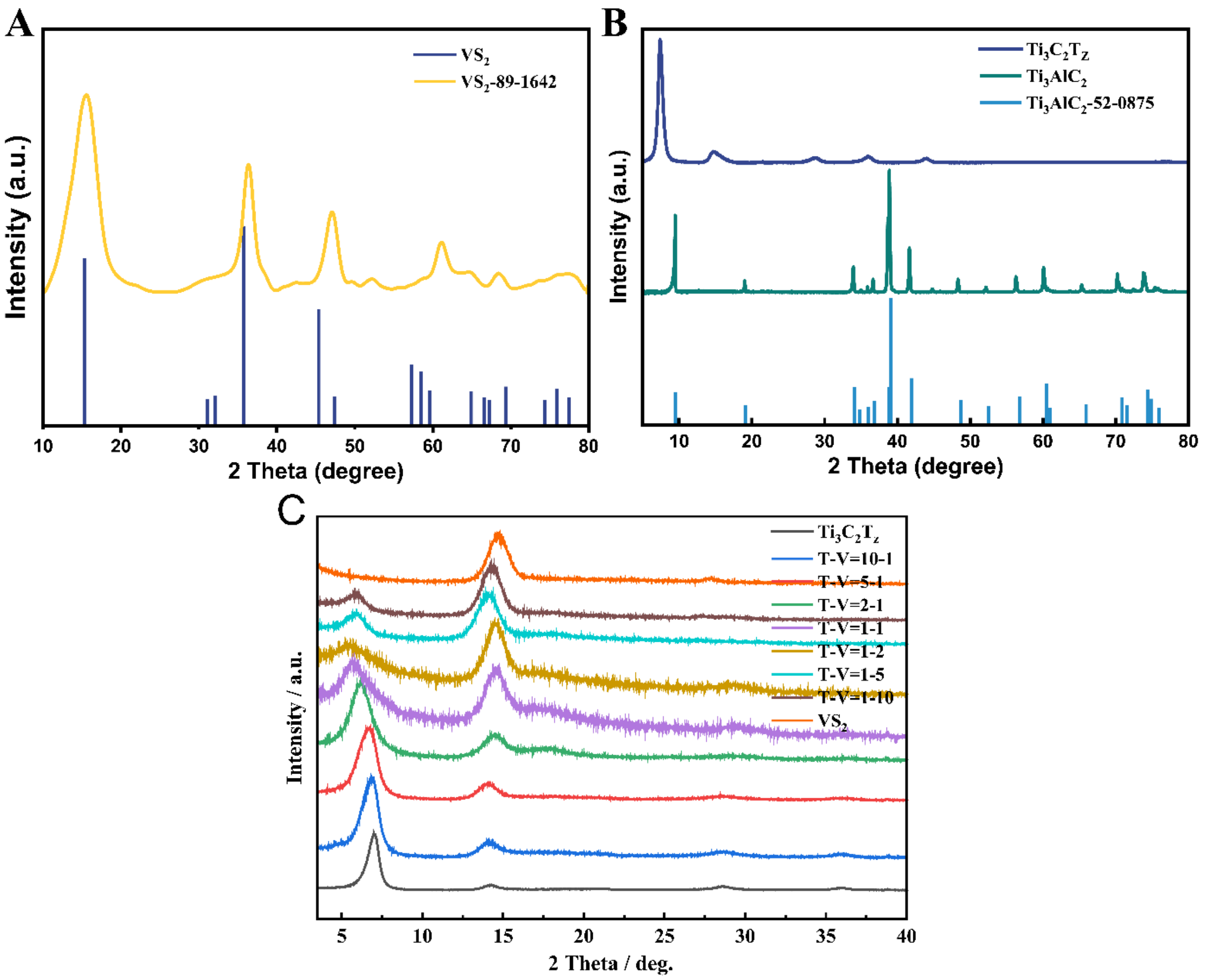

Figure S7. XRD patterns of (A) Ti_3AlC_2, $Ti_3C_2T_z$, (B) VS_2 with the standard cards and (C) $Ti_3C_2T_z/VS_2$ composite films with different mass ratios.

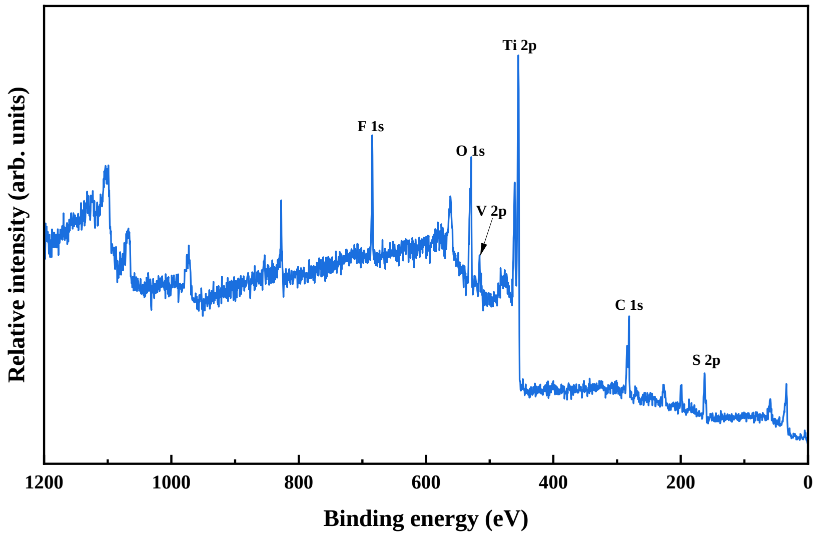

Figure S8. The XPS spectra of the T-V=1:5 free-standing film.

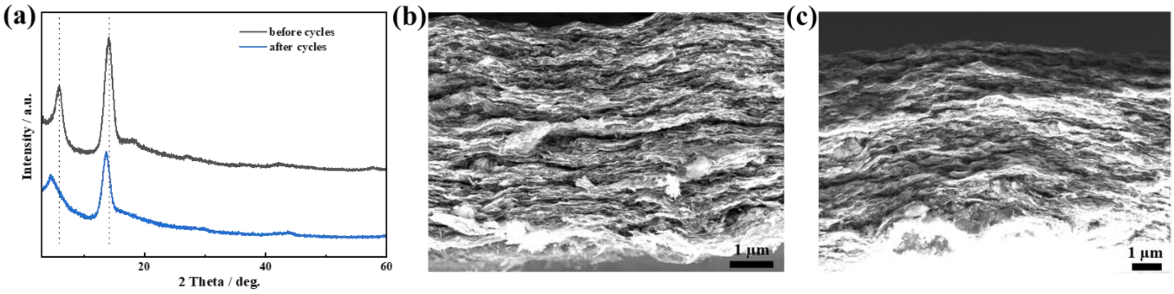

Figure S9. (a) XRD patterns of T-V=1:5 electrode before and after 6000 cycles. Cross-sectional SEM image of T-V=1:5 electrode before (b) and after (c) 6000 cycles.

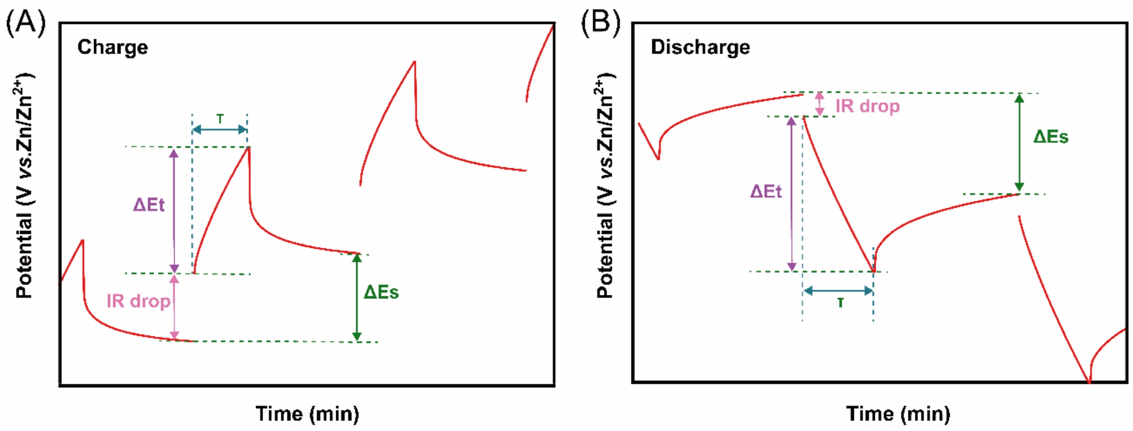

Figure S10. Schematic for GITT in a) Discharge, and b) Charge. (τ is relaxation time, ΔEs voltage change due to current pulse, and ΔEτ voltage change during current pulse (eliminating voltage change following relaxation time).

Scan rate, *v* (mV s^{-1})	Peak current, I^+ (mA)
2	0.75
3	0.8779
4	1.054
5	1.247
6	1.4
7	1.559
8	1.707
9	1.846
Mass of active material: 0.3 mg	

Figure S11. The oxidation peak values correspond to different scan rate in the CV curve of T-V=1:5.

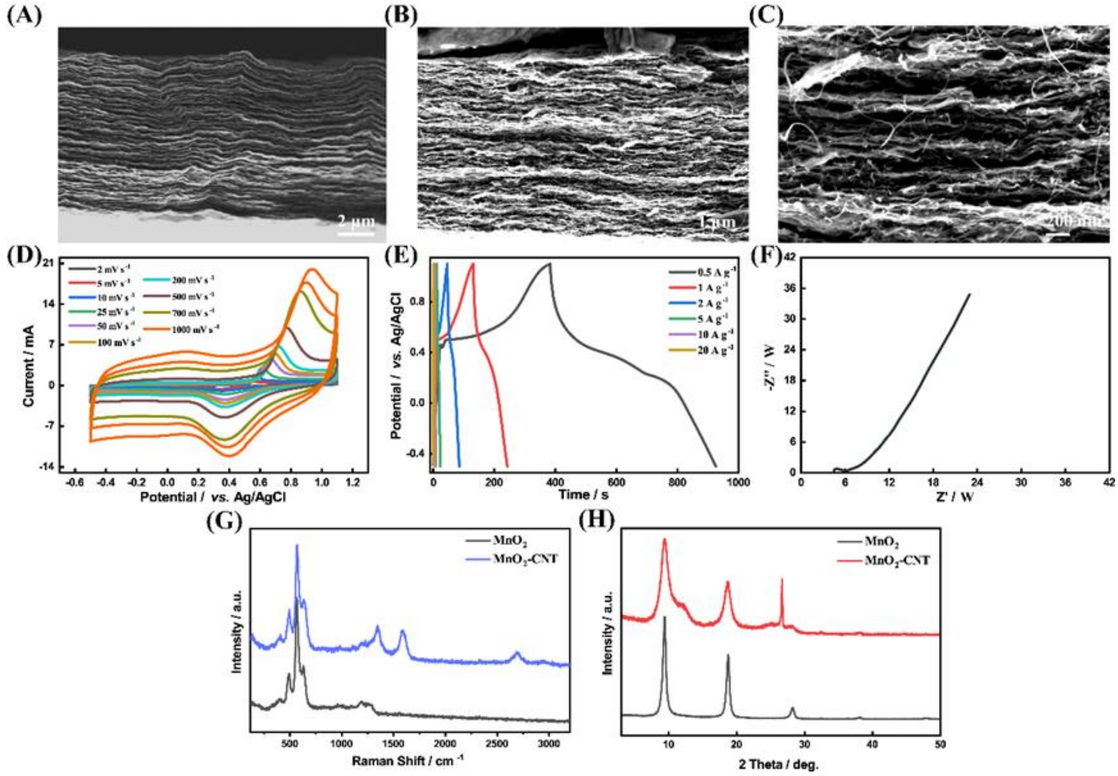

Figure S12. Cross-sectional SEM image of (A) MnO_2 at scale bars of 2 μm, (B) MnO_2-CNT at scale bars of 1 μm, (C) MnO_2-CNT at scale bars of 200 nm. (D) CV curves of MnO_2-CNT at different scan rates. (E) Galvanostatic charge/discharge (GCD) curves of MnO_2-CNT at different current densities. (F) Nyquist plot of MnO_2-CNT tested at the open-circuit potential within the frequency range from 10^{-2} to 10^5 Hz.

Table S1. Summary of global atomic percentages obtained from XPS survey spectra for T-V=1:5 film.

	V at.%	S at.%	Ti at.%	C at.%	O at.%	F at.%
T-V=1:5	10.2	26.1	6.5	40.8	10.8	5.6

Table S2. The comparison of various vanadium dioxide-based and MXene-based materials and their electrochemical performances in AZIBs.

**Materials**	**Electrolyte**	**Voltage Window**	**Cycling Stability**	**Reference**
V_2O_5	3 M $ZnSO_4$	0.2–1.6 V	85.3% capacity retention at 5 A g^{-1} after 1500 cycles	[2]
V_2O_5/C	15 M LiTFSI + 1 M Zn(CF3SO$_3$)$_2$	0.2–1.6 V	90.7% capacity retention at 5 A g^{-1} after 1500 cycles	[2]
TiCx@VO_2	2 M ZnOTf	0.4-1.6 V	82% capacity retention at 20 A g^{-1} after 2600 cycles	[3]
MXene@ VO_2	2 M ZnOTf	0.2-1.4 V	76% capacity retention at 1 A g^{-1} after 5000 cycles	[4]
VN@Ti_3C_2	3 M $ZnSO_4$	0.1–1.6 V	82.8% capacity retention at 5 A g^{-1} after 2000 cycles	[5]
MoO_{3-x}@Ti_3C_2	2 M $Zn(CF_3SO_3)_2$	0.25–1.3 V	46.7% capacity retention at 4 A g^{-1} after 1600 cycles	[6]
V_2O_5 /$Ti_3C_2T_x$	3 M $Zn(CF_3SO_3)_2$	0.2–1.6 V	99.5% capacity retention at 10 A g^{-1} after 5000 cycles	[7]
VS_2/$V_4C_3T_x$	3 m $Zn(CF_3SO_3)_2$	0.2–1.5 V	88.8% capacity retention at 5 A g^{-1} after 800 cycles	[8]
V_2CT_x	21 M LiTFSI + 1M Zn(CF3SO$_3$)$_2$	0.2–2.0 V	89.1% capacity retention at 5 A g^{-1} after 4000 cycles	[9]
VS_2/V_2CT_x	3 m $Zn(CF_3SO_3)_2$	0.2–1.6 V	90% capacity retention at 10 A g^{-1} after 60 cycles	[10]
VO_2@V_2C	3 M $Zn(CF_3SO_3)_2$	0.2-1.2 V	82% capacity retention at 0.2 A g^{-1} after 100 cycles	[11]
V_2O_x@V_2CT_x	1 M $ZnSO_4$	0.3-1.6 V	81.6% capacity retention at 1 A g^{-1} after 200 cycles	[12]
V_2O_5@V_2C	3 M $Zn(CF_3SO_3)_2$	0.2-1.2 V	82% capacity retention at 0.2 A g^{-1} after 100 cycles	[13]
VSe_2@V_2CT_x	2 M $Zn(CF_3SO_3)_2$	0.2–1.6 V	93.1% capacity retention at 2 A g^{-1} after 600 cycles	[14]
$CeVO_4$/V_2	3 M $ZnSO_4$	0.2–1.6	81.6% capacity retention at	[15]

CT_x		V	5 A g^{-1} after 3000 cycles	
Nb_2CT_x	21 M LiTFSI + 1 M $Zn(OTf)_2$	0.1–2.4 V	89% capacity retention at 0.5 A g^{-1} after 1500 cycles	[16]
Nb_2O_5@$Nb_4C_3T_x$	1 M $LiClO_4$-EC/DMC	0.1–1.8 V	100% capacity retention at 2 A g^{-1} after 400 cycles	[17]
**$VS_2/Ti_3C_2T_z$**	**1 M $ZnSO_4$**	**0-2.0 V**	**96.7% capacity retention at 2 A g^{-1} after 5000 cycles**	**This work**

Reference:

[1] M. Alhabeb, K. Maleski, B. Anasori, P. Lelyukh, L. Clark, S. Sin, Y. Gogotsi, *Chem. Mater.* **2017**, *29*, 7633.

[2] H. Liu, L. Jiang, B. Cao, H. Du, H. Lu, Y. Ma, H. Wang, H. Guo, Q. Huang, B. Xu, S. Guo, *ACS Nano.* **2022**, *16*, 14539.

[3] T. Wang, S. Li, X. Weng, L. Gao, Y. Yan, N. Zhang, X. Qu, L. Jiao, Y. Liu, *Adv. Energy Mater.* **2023**, *13*, 2204358.

[4] P. Liang, K. Zhu, J. Chen, Y. Rao, H. Zheng, J. Guo, Z. Kong, J. Zhang, J. Liu, K. Yan, J. Wang, *J. Power Sources.* **2023**, *568*, 232945.

[5] Z. Xu, X. Li, Y. Jin, Q. Dong, J. Ye, X. Zhang, Y. Qian, *Nanoscale.* **2022**, *14*, 11655.

[6] W. Du, L. Miao, Z. Song, X. Zheng, Y. Lv, D. Zhu, L. Gan, M. Liu, *J. Power Sources.* **2022**, *536*, 231512.

[7] J. Shi, Y. Hou, Z. Liu, Y. Zheng, L. Wen, J. Su, L. Li, N. Liu, Z. Zhang, Y. Gao, *Nano Energy.* **2022**, *91*, 106651.

[8] Y. Mao, J. Bai, S. Lin, P. Wang, W. Li, K. Xiao, S. Wang, X. Zhu, B. Zhao, Y. Sun, *Small.* **2023**, e2306615.

[9] X. Li, M. Li, Q. Yang, G. Liang, Z. Huang, L. Ma, D. Wang, F. Mo, B. Dong, Q. Huang, C. Zhi, *Adv. Energy Mater.* **2020**, *10*, 2001791.

[10] F. Yu, Z. Yang, X. Zhang, P. Yang, L. Li, J. Ma, *Journal of Materials Chemistry A.* **2022**, *10*, 23531

[11] J. Chen, B. Xiao, C. Hu, H. Chen, J. Huang, D. Yan, S. Peng, *ACS Appl. Mater. Interfaces.* **2022**, *14*, 28760.

[12] R. Venkatkarthick, N. Rodthongkum, X. Zhang, S. Wang, P. Pattananuwat, Y. Zhao, R. Liu, J. Qin, *ACS Appl. Energy Mater.* **2020**, *3*, 4677.

[13] D. Sha, C. Lu, W. He, J. Ding, H. Zhang, Z. Bao, X. Cao, J. Fan, Y. Dou, L. Pan, Z. Sun, *ACS Nano.* **2022**, *16*, 2711.

[14] W. Xu, X. Zhang, J. Li, X. Chen, L. Lan, J. Zhang, F. C.-C. Ling, Q. Ru, *Ionics.* **2023**, *1*, 11.

[15] X. Li, X. Ma, Y. Hou, Z. Zhang, Y. Lu, Z. Huang, G. Liang, M. Li, Q. Yang, J. Ma, N. Li, B. Dong, Q. Huang, F. Chen, J. Fan, C. Zhi, *Joule.* **2021**, *5*, 2993.

[16] C. Zhang, S. J. Kim, M. Ghidiu, M. Q. Zhao, M. W. Barsoum, V. Nicolosi, Y. Gogotsi, *Adv. Funct. Mater.* **2016**, *26*, 4143.

[17] F. Long, Q. Zhang, J. Shi, L. Wen, Y. Wu, Z. Ren, Z. Liu, Y. Hou, K. Mao, K. Niu, *Chem. Eng. J.* **2023**, *455*, 140539.
